# Antiinflammatory and Antiphotodamaging Effects of Ergostatrien-3β-ol, Isolated from *Antrodia camphorata*, on Hairless Mouse Skin

**DOI:** 10.3390/molecules21091213

**Published:** 2016-09-10

**Authors:** Yueh-Hsiung Kuo, Tzu-Yu Lin, Ya-Jhen You, Kuo-Ching Wen, Ping-Jyun Sung, Hsiu-Mei Chiang

**Affiliations:** 1 Department of Chinese Pharmaceutical Sciences and Chinese Medicine Resources, China Medical University, Taichung 404, Taiwan; kuoyh@mail.cmu.edu.tw; 2 Department of Biotechnology, Asia University, Taichung 413, Taiwan; 3 Department of Cosmeceutics, China Medical University, Taichung 404, Taiwan; fishlin522@gmail.com (T.-Y.L.); jenn123gay@yahoo.com.tw (Y.-J.Y.); kcwen0520@mail.cmu.edu.tw (K.-C.W.); 4 National Museum of Marine Biology and Aquarium, Pingtung 944, Taiwan; pjsung@nmmba.gov.tw

**Keywords:** ergostatrien-3β-ol, photodamage, hyperplasia, erythema, nuclear factor kappaB, transepidermal water loss

## Abstract

Ergostatrien-3β-ol (EK100), isolated from the submerged whole broth of *Antrodia camphorata*, has antidiabetic, hyperlipidemic, and hepatoprotective activities. However, the antiphotodamage activity of EK100 has still not been revealed. Inflammation and collagen degradation contribute to skin photodamage and premature aging. In the present study, in vivo experiments were designed to investigate the antiinflammatory and antiphotodamaging activities of EK100 in hairless mice by physiological and histological analysis of the skin. Results indicated that topical application of EK100 (25 and 100 μM) for 10 weeks efficiently inhibited ultraviolet B (UVB)-induced wrinkle formation, erythema, and epidermal thickness in the mice skin. EK100 also restored UVB-induced collagen content reduction in hairless mice skin. In addition, the immunohistochemistry results indicated that EK100 significantly inhibited the UVB-induced expression of matrix metalloproteinase-1 (MMP-1), interleukin-6 (IL-6), inducible nitric oxide synthase (iNOS), and nuclear factor kappaB (NF-κB) in the mouse skin. The expression of these proteins was similar to the Normal group after 100 μM EK100 treatment. EK100 inhibited collagen degradation in the skin through MMP-1 inhibition and antiinflammation. EK100 significantly reduced the transepidermal water loss (TEWL), indicating that EK100 protected skin from UVB-induced damage. Our findings strongly suggest that EK100 has significant beneficial antiinflammatory and antiphotoaging activities and that EK100 can be developed as an antiphotodamaging agent.

## 1. Introduction

The skin is the outermost tissue of the human body and is easily affected by environmental factors, such as solar radiation, smoking, and pollution. Skin aging is a continual and complex process, and many factors are involved in this process. Photoaging is attributed to continuous exposure to ultraviolet (UV). The characteristics of skin aging include coarse wrinkles, hyperplasia, dryness, laxity and hyperpigmentation [1]. Chronic UV exposure increases the number of stratum corneum layers and keratinocytes expressing filaggrin, which is a marker of terminal differentiation [2,3]. In addition, repeated exposure to sunlight causes damage to collagen fibers and an excessive deposition of abnormal elastic fibers [4,5]. Finally, this type of exposure increases epidermal thickness and reduces skin elasticity, causing skin aging and damage [6,7,8].

Chronic UV exposure causes serious skin injuries by generating oxidative stress, inflammation, and DNA lesions [9,10,11]. UV exposure increases the generation of reactive oxygen species (ROS) causing peroxidation of the cell membrane, subsequently leading to the damage of keratinocytes and fibroblasts. ROS triggers expression of matrix metalloproteins (MMPs) and genes, such as MMP-1 and MMP-3, causing photodamage, including wrinkle formation, solar elastosis, and extracellular matrix (ECM) degradation such as collagen, elastin, and proteoglycan, which maintain the cell and skin structures [12,13]. UV irradiation upregulates inflammation-related genes, including interleukin (IL)-1 and -6 receptors; and peroxisome proliferator-activated receptor gamma, and cause release of proinflammatory cytokines such as IL-1, IL-6 and TNF-α. Furthermore, UV also increases the prostaglandin E_2_ concentration and generating nitric oxide (NO) [9,14]. The overexpression of inducible NO synthase (iNOS) and translocation of nuclear factor kappaB (NF-κB) into the nucleus have been reported to trigger downstream signaling transduction, thus causing skin inflammation [15,16].

Ergostatrien-3β-ol (EK100) was isolated from the submerged whole broth of *Antrodia camphorata (A. camphorata)*, which is a widely used herb. Because of its rarity and difficult cultivation, *A. camphorata* is a highly valued polypore mushroom that is native only to Taiwan [17,18]. The fruiting body and cultured mycelia are composed of fatty acids, lignans, sesquiterpenes, and triterpenoids [19]. Furthermore, the mycelia from submerged culture is used in health food and nutraceuticals [20]. The fermented cultured broth has shown antioxidant, antiinflammatory, vasodilatory, and hepatoprotective properties [21,22,23]. EK100, a major component of the submerged whole broth of *A. camphorata*, was reported to have antidiabetic and dyslipidemic activities in high-fat-diet-fed mice [17]. In addition, EK100 exhibited antiinflammatory effects on λ-carrageenan-induced paw edema in mice [24]. 

Compounds or natural products such as polyphenols and anthocyanin that exhibit an antioxidant or antiinflammatory activity or inhibit MMPs expressions have been shown to protect the skin against photoaging and photocarcinogenesis [1,25,26]. Therefore, EK100 with strong antioxidant and antiinflammation activity may be a desirable candidate for further development as a treatment for UV-induced skin inflammation and damage. In this study, we focused on antiphotodamage activity of EK100, particularly its antiinflammatory and antiphotoaging activities and the associated mechanisms on hairless mouse skin.

## 2. Results

### 2.1. Body Weight of the Mice with Topical Application of EK100

**Figure 1:**
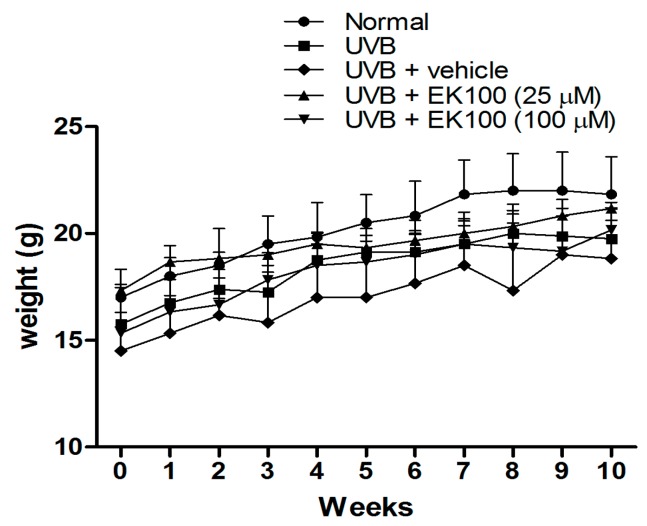
Body weight of the mice in the five groups.

All animals in each group had a similar mean body weight at baseline. The body weight of the mice in the five groups was not significantly different after the 10-week treatment (Figure 1).

### 2.2. Topical Application of EK100 Significantly Prevented UVB-Induced Skin Erythema and Damage

**Figure 2:**
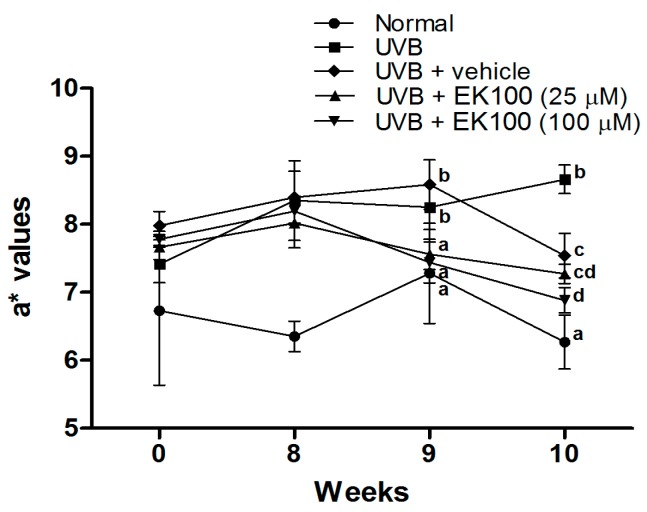
Effect of EK100 on a* values in chronic UVB-irradiated hairless mice. UVB irradiation significantly increased the a* values of the mouse skin in the 8th, 9th, and 10th weeks; however, EK100 treatment reduced the a* values in the 9th, and 10th week. ^a–d^: Values not followed by a common letter are significantly different (*p* < 0.05).

Skin erythema (a* values) indicates the extent of inflammation. In Figure 2, the results indicate that UVB irradiation significantly increased the a* values of the mouse skin in the 8th, 9th, and 10th week. EK100 treatment reduced the a* values in the 9th, and 10th week. The results suggested that topical application of EK100 inhibited UVB-induced skin erythema and inflammation.

**Figure 3:**
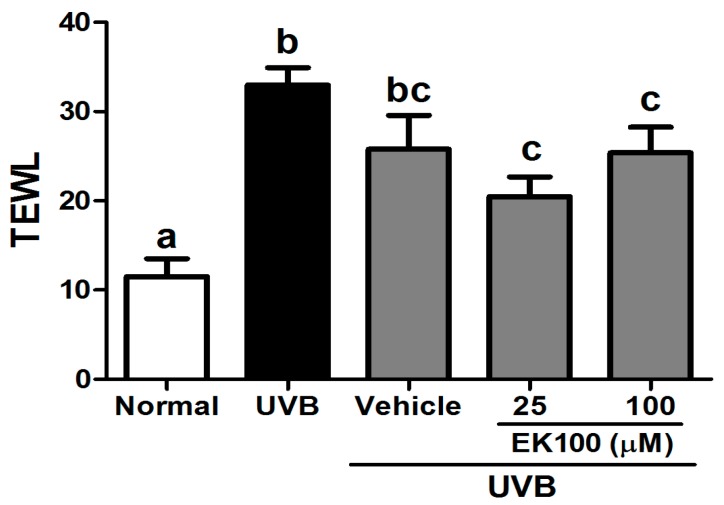
Effect of EK100 on the TEWL in UVB-irradiated hairless mice in the 10th week. UVB exposure significantly increased the TEWL in the mouse skin; however, the topical application of 25-μM and 100-μM EK100 in the mice significantly reduced the TEWL. ^a–c^: Values not followed by a common letter are significantly different (*p* < 0.05).

UVB exposure increased the TEWL, which reflected the skin barrier function, in the mouse skin. After 10 weeks of UVB exposure, the TEWL significantly increased; however, the topical application of 25 μM and 100 μM EK100 in the mice significantly reduced the TEWL (Figure 3). The results indicated that EK100 did not cause skin damage or toxicity; by contrast, it elevated the skin barrier function.

### 2.3. EK100 Prevented UVB-Induced Wrinkle Formation

**Figure 4:**
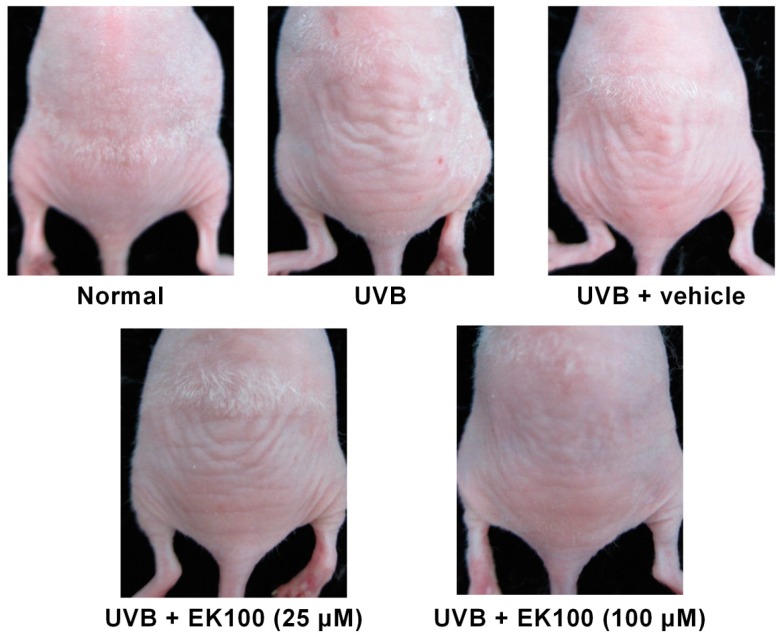
Photographs of wrinkles induced by UVB irradiation and the effect of topically applied EK100. UVB significantly increased the wrinkle formation on mouse skin, and EK100 reversed it.

Wrinkle formation was macroscopically observed and assessed in the dorsal region following the administration of UVB irradiation for 10 weeks. Ten weeks of UVB exposure significantly increased wrinkles on the dorsal skin of the mice (Figure 4). Moreover, the topical application of EK100 (25 and 100 μM) reduced wrinkle formation (Figure 4); the wrinkle score was 5.0 ± 1.0 for UVB-irradiated mice, and the score significantly decreased to 3.9 ± 1.8 and 1.6 ± 0.8 after 25 and 100 μM EK100 treatments, respectively (Table 1).

### 2.4. EK100 Reduced the Epidermal Thickness and Increased the Collagen Content in UVB-Irradiated Hairless Mouse Skin

**Figure 5:**
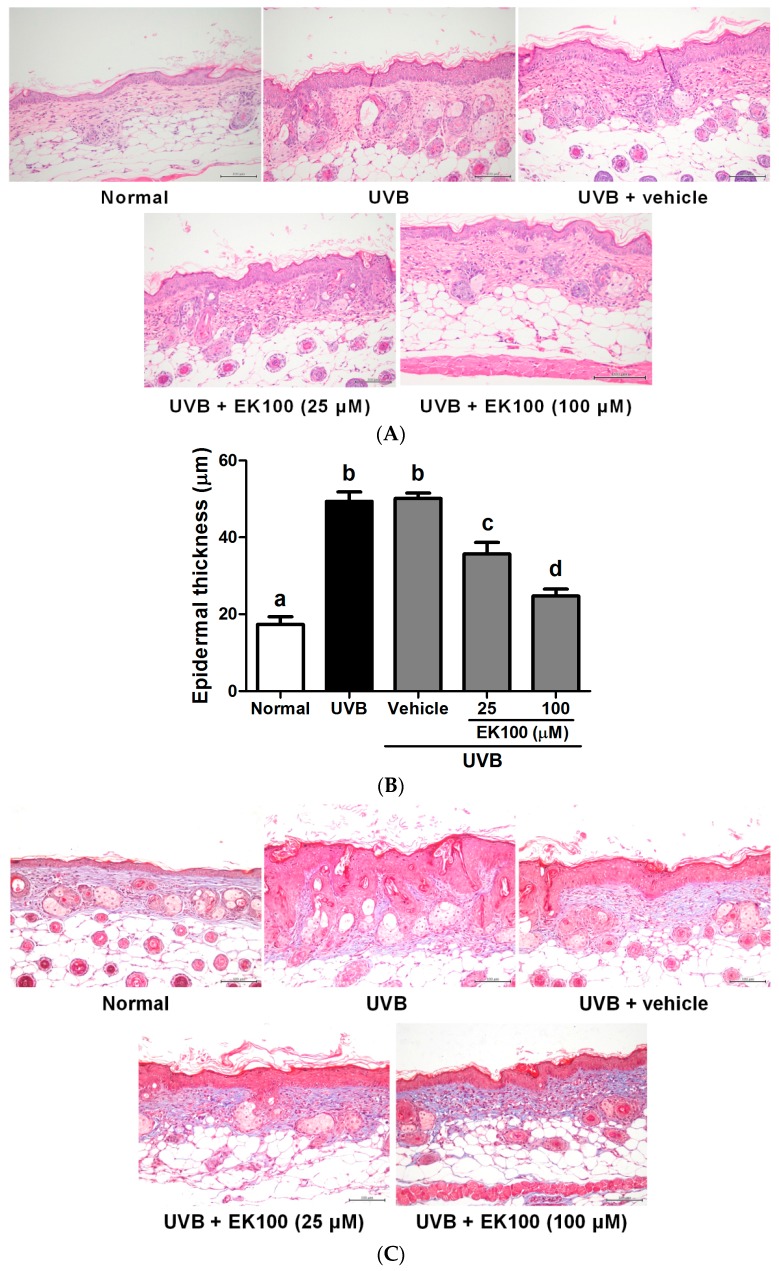
(**A**) Light micrographs of histological sections stained with hematoxylin and eosin in hairless mice; (**B**) Effect of EK100 on epidermal thickness in chronic UVB-irradiated hairless mice in the 10th week. The epidermal thickness of the mice was significantly increased by UVB exposure and reduced by EK100 treatment. ^a–d^: Values not followed by a common letter are significantly different (*p* < 0.05); (**C**) Light micrographs of histological sections stained with Masson trichrome in hairless mice. Collagen fibers were stained in blue. UVB significantly increased the thickness and reduced the collagen content in the mouse skin. EK100 reduced the epidermal thickness and increased the collagen content.

Histological examination was performed to determine the effects of EK100 on the thickness, lesion formation, and collagen content of the skin following UVB irradiation. After chronic UVB exposure, wrinkles and hyperplasia were observed in the mouse skin. The epidermal thickness of the UVB-irradiated mice significantly increased compared with that of the control mice (Figure 5A). Furthermore, the topical application of EK100 significantly inhibited the increase in the UVB-induced epidermal thickness (Figure 5A). The epidermal thickness was 17.3 ± 2.0 μm and 49.4 ± 2.4 μm in the control and UVB-irradiated groups, respectively. After the EK100 treatment, the epidermal thickness was 35.7 ± 3.0 μm in the 25-μM EK100 group and 24.7 ± 1.8 μm in the 100-μM EK100 group (Figure 5B). The epidermal thickness of the mice was significantly increased by UVB exposure and reduced by EK100 treatment. The results indicated that EK100 ameliorated the UVB-induced hyperplasia of the epidermis.

The collagen content in the mouse dorsal skin was histologically observed through Masson trichrome staining. The results indicated that the collagen content was significantly lower in the UVB-irradiated mice than in the control mice. The EK100 treatment increased the collagen content in the dermis of the mice (Figure 5C). EK100 reversed the effect of UVB on the collagen content of the skin.

### 2.5. EK100 Reduced the MMP-1 Expression in UVB-Irradiated Hairless Mouse Skin

**Figure 6:**
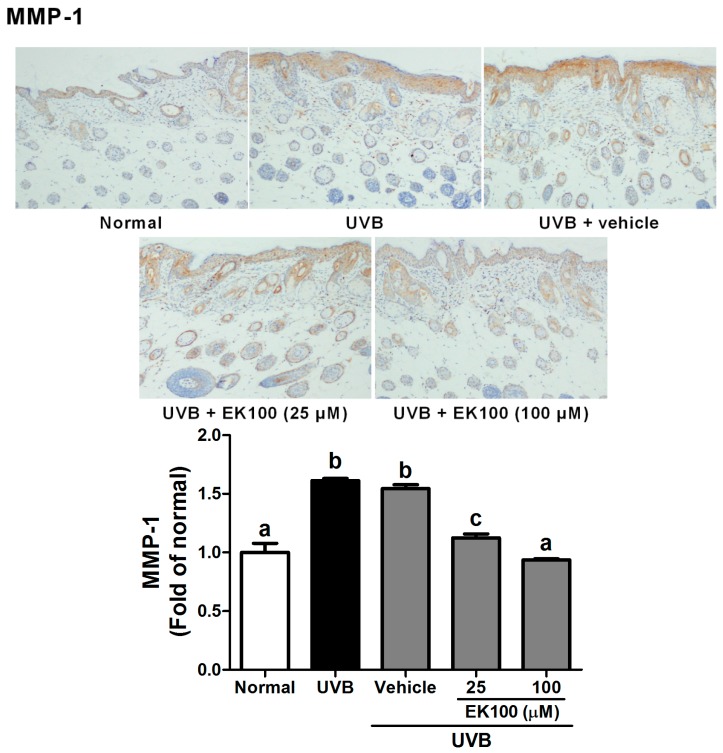
Immunohistochemical staining of MMP-1 expression in mouse skin slices. EK100 inhibited the UVB-induced overexpression of MMP-1. UVB irradiation increased the MMP-1expression and EK100 treatment reduced the MMP-1 level. ^a–c^: Values not followed by a common letter are significantly different (*p* < 0.05).

The histological effects of EK100 were examined in the skin of the UVB-irradiated hairless mice. MMP-1 is the major protein for degrading skin collagen. As shown in Figure 6, UVB irradiation increased the MMP-1expression (1.6 fold of Normal group), and EK100 treatment reduced the MMP-1 level. The MMP-1 expression was similar to that of Normal group after 100 μM EK100 treatment.

### 2.6. EK100 Reduced the Expression of IL-6, iNOS, and NF-κB in UVB-Irradiated Hairless Mouse Skin

**Figure 7:**
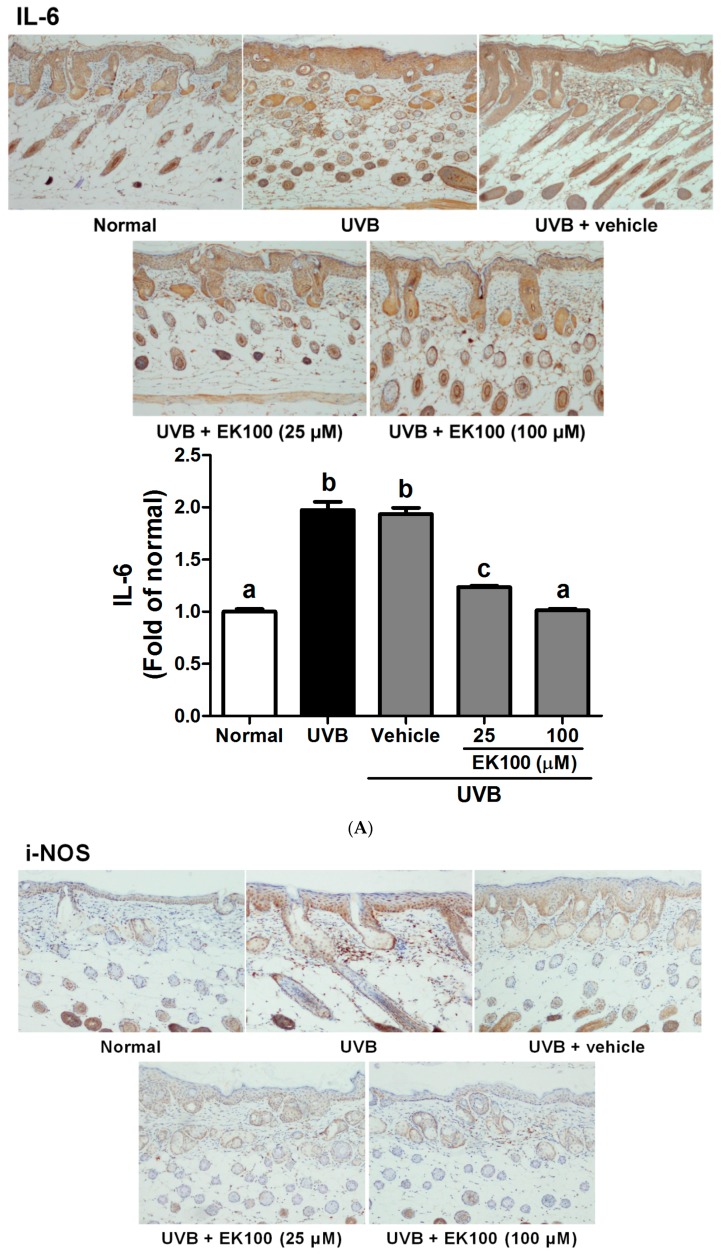

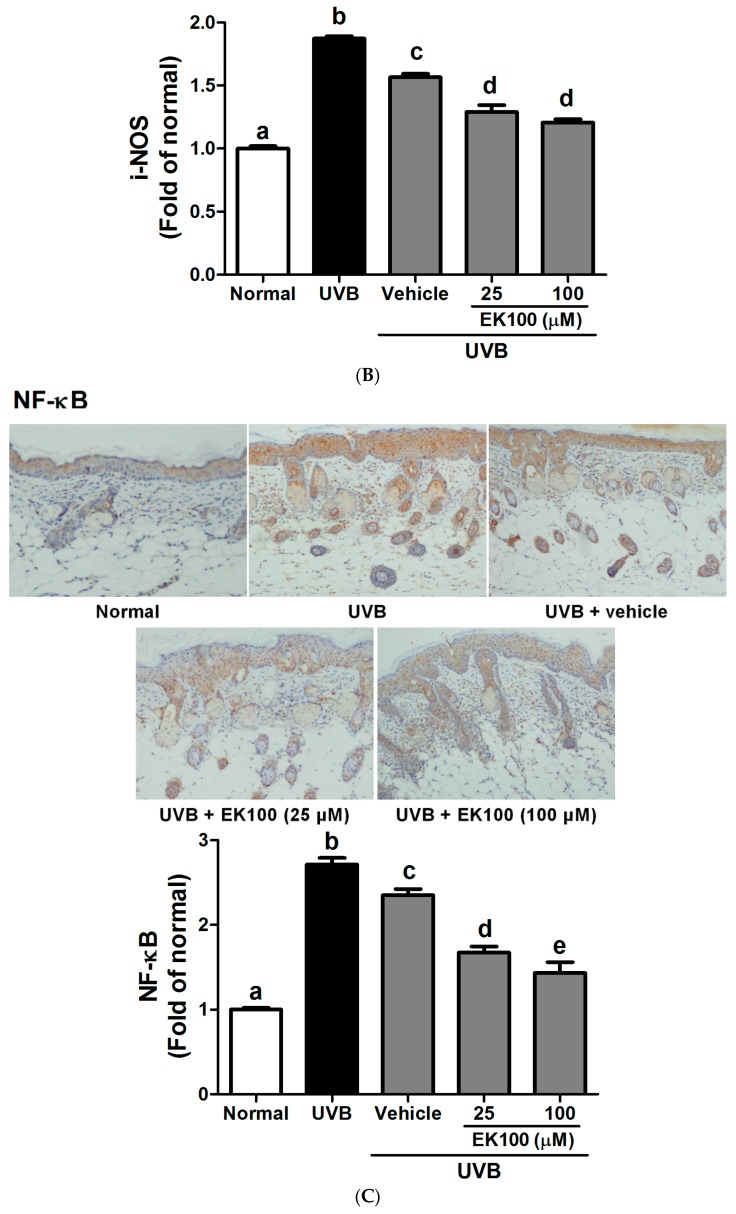
Immunohistochemical staining of IL-6 (**A**); iNOS (**B**); and NF-κB (**C**) expression in mouse skin slices. UVB irradiation increased the IL-6, iNOS, and NF-κB expression in mouse skin, while EK100 inhibited the UVB-induced overexpression of IL-6, iNOS, and NF-κB. ^a–e^: Values not followed by a common letter are significantly different (*p* < 0.05).

The production of inflammatory cytokines was examined because they are associated with UVB-induced skin and cellular damage. Figure 7A–C illustrate that UVB exposure induced the expression of IL-6, iNOS, and NF-κB and that EK100 treatment reduced the expression of these proteins. The results indicated that the UVB-induced increase in inflammation was significantly inhibited by the topical application of EK100. The expressions of IL-1 was 1.97-fold of Normal group after UVB irradiation and 1.12 and 0.94 fold after 25 and 100 μM EK100 treatment, respectively (Figure 7A). UV significantly increased iNOS expression (1.87 fold of Normal group) and EK100 reversed the effect (Figure 7B). Finally, EK100 decreased the was similar to that of Normal group after 100 μM EK100 treatment NF-κB expression from 2.71 fold to 1.43 fold of Normal group (Figure 7C). Thus, EK100 inhibited UVB-induced inflammation.

## 3. Materials and Methods

### 3.1. Materials

Paraformaldehyde and phenylmethylsulfonyl fluoride were purchased from Sigma–Aldrich Corporation (St. Louis, MO, USA). Antibodies for MMP-1, IL-6, iNOS, and NF-κB as well as β-actin were purchased from Santa Cruz Biotechnology (Santa Cruz, CA, USA). All other chemicals used in this study were of a reagent grade.

### 3.2. Isolation and Determination of the Active Compound

The freeze-dried powder of the submerged whole broth of *A. camphorata* was provided by the Biotechnology Center of Grape King Inc., Chung-Li City, Taiwan. EK100 was isolated from the *A. camphorate* freeze-dried powder and identified, as previously described [27].

### 3.3. Experimental Animals

All experimental protocols were approved by the Institutional Animal Use and Care Committee, China Medical University, Taiwan. Five-week-old BALB/cAnN.Cg-Foxn1nu/CrlNarl hairless female mice were purchased from the National Laboratory Animal Center (Taipei, Taiwan). The mice were maintained in individual ventilated caging systems under a 12-h light:dark cycle, fed standard pellet chow and water ad libitum, and housed at a controlled temperature (22 °C ± 2 °C) with humidity (50% ± 10%).

### 3.4. UVB Irradiation and Topical Application of EK100

The mice were irradiated with ultraviolet B (UVB) for 10 weeks, and all treatments were administered to the dorsal skin of the mice after UVB irradiation, as previously described [28,29,30]. The mice were randomly divided into five groups, namely Normal, UVB-irradiated, vehicle (sweet almond oil)-treated plus UVB-irradiated, UVB-irradiated plus 25 μM EK100-treated, and UVB-irradiated plus 100 μM EK100-treated groups. Each group had at least 6 mice. The UVB-irradiated mice were treated with 36 mJ/cm^2^ of UVB in the first week, 54 mJ/cm^2^ of UVB in the second–fourth weeks, 72 mJ/cm^2^ of UVB in the fifth–seventh weeks, and 108 mJ/cm^2^ of UVB in the 8th–10th weeks, three times per week. Furthermore, the vehicle-treated plus UVB-irradiated mice were topically administered 50 μL of sweet almond oil daily and the EK100-treated mice were topically administered an equal volume of sweet almond oil containing 25 μM or 100 μM EK100 daily. The Normal mice remained untreated. The degree of wrinkle formation was assessed according to the grading scale described in previous studies, and the name of the group was unrevealed to the researcher [29,30]. After the experiments, the mice were sacrificed, and the dorsal skin was excised for histopathological studies.

### 3.5. Measurement of Skin Erythema and Transepidermal Water Loss

To examine the effects of EK100 on UVB-induced erythema and transepidermal water loss (TEWL), the erythema was detected in the 8th, 9th, and 10th week by using a spectrocolorimeter, and the TEWL was measured in the 10th week by using Tewameter^®^ TM 300 (Courage + Khazaka electronic GmbH, Cologne, Germany).

### 3.6. Skin Hyperplasia and Collagen Degeneration

The skin samples were fixed in 10% formalin and embedded in paraffin. Vertical sections were cut, mounted on a glass slide, and stained with either hematoxylin and eosin or Masson trichrome. The thickness of each epidermal section from the basal layer to the stratum corneum was measured using a microscope equipped with an ocular micrometer, as previously described [29]. The skin thickness of each section was determined by Image J software (Wayne Rasband, National Institutes of Health, Bethesda, MD, USA).

### 3.7. Immunohistochemical Staining

The skin samples were fixed in 10% formalin, embedded in paraffin, and sectioned at 5 μm. The paraffin sections were stained with the following monoclonal antimouse antibodies: MMP-1, IL-6, iNOS, and NF-κB (Santa Cruz). Histopathology was examined through microscopy and the density of MMP-1, IL-6, iNOS, and NF-κB was determined by Image J (Wayne Rasband, National Institutes of Health.

### 3.8. Data Analysis

All data in this study were expressed as mean ± standard deviation of the independent experiments performed in triplicate. The statistical analysis of the differences in means for each experimental group was performed through analysis of variance (ANOVA) followed by Tukey test in order to detect intergroup differences. *p* < 0.05 was considered statistically significant. 

## 4. Discussion

UV irradiation is the main environmental hazard for causing skin damage and aging. UV exposure generates ROS, resulting in skin disorders, including DNA damage, inflammation, and even skin cancer [31]. UVB-mediated skin damage is correlated with the production of inflammatory mediators in epidermal keratinocytes, including IL-1β and IL-6 [10,32,33]. UV can cause release of IL-1 from the stratum corneum to initiate inflammation within the living layers of skin, and induce synthesis and release of other proinflammatory cytokines [34]. IL-6 is one of the cytokines that is induced in various cell types and is associated with cutaneous inflammatory disorders, such as psoriasis and photoaging [13,35]. NF-κB activation causes the expression of various gene involved in immunological and inflammatory response [36]. UV irradiation causes the nuclear translocation of NF-κB, thus inducing MMP production for degrading collagen in the human skin [37,38]. In addition, NF-κB-modulated iNOS gene transcription and protein expression cause skin inflammation [39]. Substances with an antiinflammatory activity may be antiphotodamage candidates [9,40,41,42,43,44,45]. In a previous study, the methanolic extract of *A. camphorata* was shown to protect mice against lipopolysaccharide-induced oxidative stress [46]. In the present study, EK100 suppressed UVB-induced skin erythema (a* value), an index for inflammation. To further prove the effect of EK100 on UVB-induced inflammation, the expression of inflammatory markers, namely iNOS, IL-6, and NF-κB, was determined in this study; EK100 inhibited the UVB-induced overexpression of these proteins (Figure 7). These results reveal that EK100 has an antiinflammatory activity in hairless mice skin and suggest that EK100 protects the skin from UV-induced photodamage through this activity. 

UVB is the most hazardous environmental carcinogen to humans because it generates ROS and subsequently activates downstream signaling pathways, followed by MMPs’ induction in the degradation or synthesis inhibition of collagenous ECM in connective tissues [47,48,49]. MMPs are endopeptidase capable of degrading ECM components and are involved in remodeling of dermis [1,3]. Excessive ROS and inflammatory cytokines enhance the expression and activity of MMPs [13,36,50,51]. MMP-1 is a collagenase which breaks down collagens. In addition to collagen, MMPs also degrade other ECM such as elastin, laminin and fibronectin [12]. The inhibition of MMP activities has been shown to result in the suppression of collagen degradation in the skin [4,12,42,52]. Our results indicated EK100 inhibited UVB-induced MMP-1 expression and restored the content of collagen in the hairless mice skin.

Skin photoaging is characterized by an increase in epidermal thickness and wrinkle formation and a decrease in skin elasticity, which is associated with a decrease in collagen content, the principal component of the dermal layer [4,53]. Moreover, epidermal thickness increases in response to UVB exposure for the purpose of preventing further UV damage and carcinogenesis [29,32]. Epidermal hypertrophy is widely considered a symptom of photoaging and wrinkle formation. Chronic UVB irradiation induces keratinocyte proliferation and epidermal hyperplasia, increases collagen degradation, and reduces collagen production, thereby leading to the loss of collagen and, consequently, to an increase in wrinkle formation and epidermal thickness as well as reduction in skin elasticity [29,54,55]. We observed that the topical application of EK100 ameliorated dorsal skin erythema and hyperplasia in the UVB-irradiated mice; moreover, the effect may occur by reducing the expression of MMP-1, IL-6, iNOS, and NF-κB. Moreover, UVB exposure disturbs the skin barrier function and elevation of TEWL; agents or natural products with reduced water loss or an increased natural moisturizing factor will protect the skin from UV damage [56]. The results indicated EK100 reduced the TEWL suggesting the elevation of skin barrier function by EK100.

## 5. Conclusions

**Figure 8:**
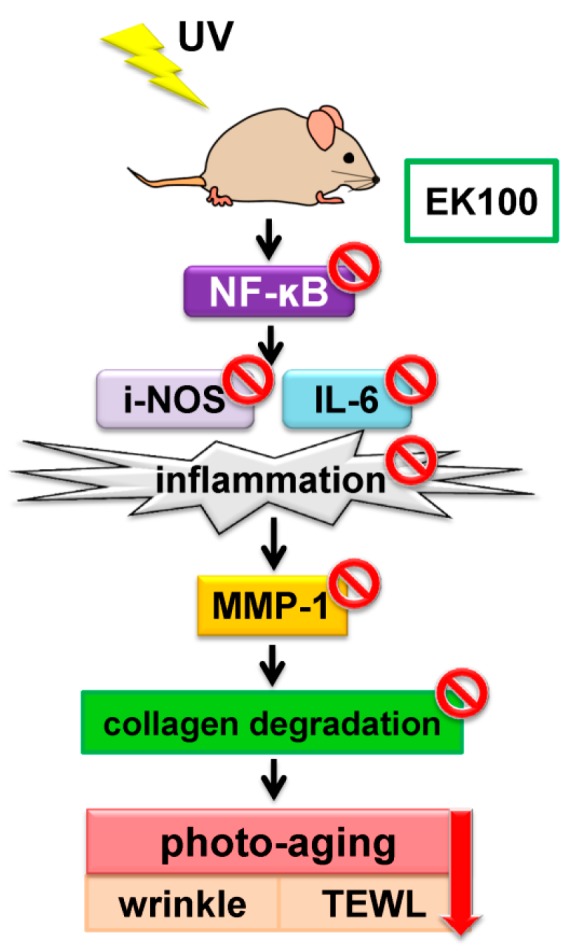
Schematic diagram of the inhibitory effects of EK100 in UVB-induced inflammation and photodamage.

The present study revealed a protective effect of topically used EK100 on UVB-irradiated hairless mouse skin by inhibiting UVB-induced inflammation, and collagen break down (Figure 8). EK100 reduced UVB-induced wrinkle formation, erythema and TEWL of mice skin. In addition, EK100 inhibited protein expression of MMP-1, IL-6, iNOS, and NF-κB induced by UVB, and subsequently suppressed the degradation of collagen in the skin. EK100 may be used in skin care in the future.

## Figures and Tables

**Table 1:** Effects of EK100 on skin wrinkles induced by UVB irradiation in hairless mice.

	Wrinkle Score
Normal mice	0.0 ± 0.0 ^a^
UVB-irradiated mice	5.0 ± 1.0 ^b^
Vehicle-treated UVB-irradiated mice	4.7 ± 1.0 ^bc^
EK100 (25 μM)-treated UVB-irradiated mice	3.9 ± 1.8 ^c^
EK100 (100 μM)-treated UVB-irradiated mice	1.6 ± 0.8 ^d^

^a–d^: Values not followed by a common letter are significantly different (*p* < 0.05).
